# Molecular Landscape of the Epithelial–Mesenchymal Transition in Endometrioid Endometrial Cancer

**DOI:** 10.3390/jcm10071520

**Published:** 2021-04-06

**Authors:** Marcin Opławski, Robert Nowakowski, Agata Średnicka, Dominika Ochnik, Beniamin Oskar Grabarek, Dariusz Boroń

**Affiliations:** 1 Department of Gynecology and Obstetrics with Gynecologic Oncology, Ludwik Rydygier Memorial Specialized Hospital, 31-826 Kraków, Poland; srednickaagata@gmail.com (A.Ś.); bgrabarek7@gmail.com (B.O.G.); dariusz@boron.pl (D.B.); 2 Department of Histology, Cytophysiology and Embryology, Faculty of Medicine, University of Technology in Katowice, 41-800 Zabrze, Poland; nowakowskirobert926@gmail.com (R.N.); dominika.ochnik80@gmail.com (D.O.); 3 District Hospital in Chrzanów, 32-500 Chrzanow, Poland; 4 Department of Nursing and Maternity, High School of Strategic Planning in Dąbrowa Górnicza, 41-300 Dąbrowa Górnicza, Poland

**Keywords:** endometrial cancer, epithelial–mesenchymal transition, tissue, liquid biopsy, miRNA, mRNA, supplementary molecular marker

## Abstract

Modern diagnostics are based on molecular analysis and have been focused on searching for new molecular markers to use in diagnostics. Included in this has been the search for the correlation between gene expression in tissue samples and liquid biological materials. The aim of this study was to evaluate the differences in the expression profile of messenger RNA (mRNA) and micro-RNA (miRNA) related to the epithelial–mesenchymal transition (EMT) in different grades of endometrial cancer (G1–G3), in order to select the most promising molecular markers. The study material consisted of tissue samples and whole blood collected from 30 patients with endometrial cancer (study group; G1 = 15; G2 = 8; G3 = 7) and 30 without neoplastic changes (control group). The molecular analysis included the use of the microarray technique and RTqPCR. Microarray analysis indicated the following number of mRNA differentiating the endometrial cancer samples from the control (tissue/blood): G1 vs. C = 21/18 mRNAs, G2 vs. C = 19/14 mRNAs, and G3 vs. C = 10/9 mRNAs. The common genes for the tissue and blood samples (Fold Change; FC > 3.0) were G1 vs. C: *TGFB1*, *WNT5A*, *TGFB2*, and *NOTCH1*; G2 vs. C: *BCL2L*, *SOX9*, *BAMBI*, and *SMAD4*; G3 vs. C *STAT1* and *TGFB1*. In addition, mRNA *TGFB1*, *NOTCH1*, and *BCL2L* are common for all grades of endometrial cancer. The analysis showed that miR-144, miR-106a, and miR-30d are most strongly associated with EMT, making them potential diagnostic markers.

## 1. Introduction

An epithelial–mesenchymal transition (EMT) is a molecular and phenotypic process of reprogramming polarized and immobile epithelial cells into mobile mesenchymal cells, the consequence of which is an increase in mobility and also invasion [1,2]. During this process, it is observed, among other things, that there is a decrease in the expression of proteins, which strengthen the adherence of cells to one another, such as E-cadherin and γ-catenin, as well as an increase in the expression of mesenchymal markers (vimentin, N-cadherin, and fibronectin) and an increase in the activity of certain extracellular matrix metalloproteinases [3,4].

This process appears in physiological conditions that condition proper embryonic development, mainly through the creation of peripheral nervous system elements, palate formation, and formation of heart valves (Type I EMT). The described process is also significant during the healing of injuries, tissue regeneration, and organ fibrosis (Type II EMT) [5,6].

In turn, type III EMT relates to cancer cells, in which genetic and epigenetic changes have occurred, in particular, in genes that code proteins connected with the progression and occurrence of distant metastasis of various malignant tumors. The initiation of EMT is essential so that the named processes can occur [7,8].

It is said that EMT is an early stage of metastasis, during which cells that underwent this process can break away from the original tumor, penetrate the basement membrane into the circulation and then convert from the mesenchymal to the epithelial phenotype. The results of which are distant metastases [9,10].

In cancer pathogenesis, the neoplastic cells in early-stage carcinomas are in an epithelial-like state and gradually acquire more mesenchymal features as tumor progression proceeds. This results in therapeutic resistance. Furthermore, when an EMT program in breast cancer cells is activated, this also leads to a tumor-initiation state, sometimes referred to as the cancer stem cells (CSC) state. This EMT-induced acquisition of stemness probably also occurs in several other types of carcinoma cells. Considering the currently available evidence, produced by many different research groups, EMT program being integral components of the malignant progression of all types of carcinoma is plausible [11,12,13].

A significant role in the regulation of gene transcriptional activity is played by epigenetic mechanisms, including micro-RNA (miRNA), that is, short 21–23 nucleotide molecules. The miRNAs are responsible for post-transcriptional gene expression regulation. Interactions between miRNA and messenger RNA (mRNA) are conditioned by the complementarity be-tween two particles [14]. MiRNAs find their usefulness as diagnostic, prognostic, and predictive markers, mainly due to their high stability as well as the resistance of these particles against ribonuclease, temperature changes, and pH as well as high bioavailability and stability [15]. MiRNA also takes part in the regulation of the expression of genes connected with EMT. For example, miR-29b, through the regulation of EMT signalization, halts metastases in breast cancer [16], whereas miR-148a can negatively regulate Met/Snail signalization and prevent EMT and metastasis of liver cancer cells [17]. It is indicated that the EMT process relates to miR-200a, which lowers the expression of E-cadherin with a simultaneous increase in the transcriptional activity of vimentin by directly influencing the mRNA *FOXA2* [18]. In turn, it seems that miR-21-5p can also significantly affect the EMT process, which accompanies endometrial cancer [19].

Modern oncological diagnosis is based on the liquid biopsy method. The method is based on taking liquid biological material (blood, plasma, serum, washings) to carry out molecular analysis. In reference to cancer, the use of liquid biopsy is based on the fact that cancer cells release nucleic acid fragments into the bloodstream [20].

Endometrial cancer is one of the most commonly occurring gynecological cancer worldwide. The Global Cancer Observatory (GLOBOCAN) database indicates that in 2020, 604,127 new cases were diagnosed, which is approximately 13.3% of all gynecological cancer cases [21].

We distinguish the following degrees of pathomorphological differentiation: G1, highly differentiated (<5% solid cancer); G2, medium differentiated (6–50% solid cancer); and G3, poorly differentiated (>50% solid cancer). Furthermore, Type I estrogen-dependent carcinomas include G1/2 endometrial cancers. On the other hand, type II includes endometrial G3, serous, clear cell tumors, squamous, mucous, meso-nephroid, and low-differentiated tumors [22,23].

Molecular diagnosis of endometrial cancer is part of the modern diagnostic and therapeutic strategy of personalized medicine. It is worth considering that molecular changes occur before phenotypic changes. In turn, the use of hierarchal clustering with Euclidean distance in microarray analysis allows for the determination if the sample described in the examination, described as histopathological advancement stage G1, is also molecularly of the same advancement stage G1 or higher. In this respect is the research of Talkou et al., who presented the Proactive Molecular Risk Classifier for Endometrial Cancer (ProMisE), a molecular classification system based on The Cancer Genome Atlas genomic subgroups. The authors compared the ability of ProMisE with that of current risk-stratification systems (European Society of Medical Oncology (ESMO)). The use of molecular profiling in a significant way contributes to the choice of the most effective treatment option, including molecular-target therapy, as well as allowing for a precise determination of the prognosis [24].

The aim of this study was the assessment of the expression profile of mRNA and miRNA connected with the epithelial–mesenchymal transition in biopsies and the blood of endometrial cancer patients (G1–G3), compared to a control group as well as the search for the relationship between the expression of marked transcripts in tissue and blood.

## 2. Materials and Methods

The study was performed in accordance with the guidelines of the 2013 Declaration of Helsinki on human experimentation. Data confidentiality and patient anonymity were maintained at all times. Patient-identifying information was deleted before the database was analyzed. It is not possible to identify patients on an individual level either in this article or in the database. Informed consent from all patients was obtained. Approvals of the Bioethical Committee operating at the Regional Medical Chamber in Krakow, nos. 185/KBL/OIL/2020 and 186/KBL/OIL/2020, were obtained for this study.

### 2.1. Patients

The study included 30 women who qualified for hysterectomy: 30 with a diagnosed endometrioid endometrial adenocarcinoma (study group) and 30 who did not show neoplastic changes during routine gynecological examinations (control group).

All samples from a study and a control group were from histopathological examination before a molecular analysis.

Both the study and control groups consisted of patients above 45 years of age and after the childbearing period, who were being treated at the Department of Gynecology and Obstetrics with Gynecologic Oncology at the Ludwik Rygier Memorial Specialized Hospital. The study group consisted of patients with a diagnosed endometrial cancer. The diagnosed cancer was determined before the patients were recruited for the study, based on the result of the histopathological examination, obtained during diagnostic abrasion at after the hysteroscopic examination. In all cases surgery was performed, which included the radical removal of the uterus and removal of the pelvic and preaortic lymph nodes. Such course of action is the gold standard in the treatment of this cancer.

Exclusion criteria from the study group were as follows: endometriosis or adenomyosis, non-endometrioid endometrial cancer, adenocarcinoma with squamous elements, coexisting cervical carcinoma, the use of hormone therapy 24 months before surgery, extreme obesity (BMI > 40), and actual or previous history of other types of cancers. The histopathological assessment of endometrial tissue samples allowed for the division of the study group according to the degree of histological differentiation: G1 (well-differentiated), 15 cases; G2 (moderately differentiated), eight cases; and G3 (poorly differentiated), seven cases (Table 1).

### 2.2. Materials

From each patient, belonging to either the study group or control group, during hysterectomy a section of the endometrium was taken for molecular analysis and stored in Allprotect Tissue Reagent (Qiagen, Cat No./ID: 76405) Eppendorf tube, where it was stored at a temperature of −20 °C until molecular analysis was begun.

Furthermore, on the day of the operation, from each patient belonging to either the study group or control group, whole blood was extracted into PAXgene RNA kit tubes, where they were stored at a temperature of −20 °C until molecular analysis was begun.

### 2.3. RNA Isolation

The total ribonucleic acid (RNA) extraction from the tissues was carried out using the TRIzol reagent (INvitrogen Life Technologies, Carlsbad, CA, USA, catalog number 15596026) following the manufacturer’s protocol.

In turn, RNA extraction of RNA was made using the commercially available PAXgene Blood RNA kit (Qiagen, Cat No./ID: 762174) and PAXgene Blood miRNA kit (Qiagen, Cat No./ID: 763134) per the manufacturer’s protocol.

The RNA extracts were evaluated through the analysis of 18S ribosomal RNA (rRNA) and 28S rRNA (agarose electrophoresis) and by analyzing RNA concentration (260 nm) and purification (absorbance ratio 260 nm/280 nm). For the microarray analysis, RNA extracts were qualified if they had an absorbance ratio value 260 nm/280 nm in the range 1.8–2.0.

### 2.4. The mRNA Microarray Analysis

The evaluation of the expression pattern of mRNAs connected with the EMT was done using oligonucleotide microarray microarrays HG-U133A 2_0 (Affymetrix, Santa Clara, CA, USA), the GeneChip™ 3′IVT PLUS Reagent Kit, and GeneChip™ HT 3′IVT PLUS Reagent Kit (ThermoFisher, Catalog Number 902416) according to the manufactures’ protocol. The names of the probes and their ID number were obtained from the Affymetrix NetAffx™ Analysis Center database after entering the phrase “EMT” (http://www.affymetrix.com/analysis/index.affx; accessed on 25 November 2020). Data were analyzed via using microarray scanning GeneArray scanner (Agilent Technologies, Santa Clara, CA, USA).

### 2.5. The miRNA Microarray Analysis

In reference to the analyzed genes, a predictive assessment of the influence on their miRNA molecules was carried out. To do this, the microarray miRNA GeneChip miRNA 2.0 Array (Affymetrix) was used according to the manufacture′s protocol. GeneArray Scanner 3000 7G (Agilent Technologies) was used for scanning of the microarray.

To determine which of the differentiating miRNAs of the endometrial cancer samples G1–G3 in comparison to the control samples could potentially affect the expression pattern of the differentiating mRNAs, the miRNA target prediction tools mirTAR (http://mirtar.mbc.nctu.edu.tw/human/predictionIndex.php; accessed on 25 November 2020) was used.

### 2.6. Reverse-Transcription Quantitative Polymerase Chain Reaction

In the next stage of molecular analysis, the RTqPCR reaction was carried out to validate the microarray data. RTqPCR validation of array data was performed on the same sample used for the microarray. This was completed using the SensiFast ™ SYBR No-ROX One-Step Kit (Bioline, London, UK), where β-actin was used as the endogenous control.

The thermal profile of the reaction was as follows: reverse transcription (45 °C, 10 min), activation of the polymerase (95 °C, 2 min), 40 cycles including denaturation (95 °C, 5 s), annealing (60 °C, 10 s), and elongation (72 °C, 5 s).

If the value of Fold Change (FC) is higher than 1.0, it means, compared to the control culture, that there is an overexpression of genes, whereas, if the FC value is lower than 1.0, compared to the control, there is a downregulation of genes. The sequence of primers is presented in Table 2.

### 2.7. Western Blot Analysis

To separate the protein from the endometrial cancer tissues G1–G3 as well as from the samples taken from the control group patients, RIPA Lysis Buffer (EURx, Poland, catalog number E0295) was added. The cells were lysed on ice for 1 h and then the lysate was centrifuged (10 min, 4 °C, 10,000× *g*). The supernatant containing the proteins was transferred to 1.5-mL tubes and stored at −80 °C. Protein detection was performed through incubation with the appropriate primary and secondary antibodies (Santa-Cruz Biotechnology, Santa Cruz, CA, USA). To analyze the level of selected proteins, the following antibodies were utilized: Primary Anti-TGF beta 1 antibody [EPR21143] (ab215715) at a dilution of 1:1000, Anti-Notch1 antibody [mN1A] (ab128076)) at a dilution of 1:1000, Anti-Bcl2L2 antibody (ab190952) at a dilution of 1:1000, primary and secondary anti-HRP antibodies conjugated with horseradish peroxidase at a dilution of 1:5000. Antibodies were purchased from Abcam, USA.

The antibody against β-actin was conjugated with horseradish peroxidase, so it did not require a secondary antibody. Next, the same procedure as described previously was followed based on densitometric analysis using the Kodak MI 4.5SE software. The results of TGFβ1, NOTCH1, and BCL2L2 expression were normalized against the β-actin protein and shown as relative optical density.

### 2.8. Statistical Analysis

Statistical analysis of the obtained results was done using the Transcriptome Analysis Console program (Thermo Fisher Scientific, Waltham, MA, USA) and STATISTICA 13 PL software (Cracow, Poland). The ANOVA variance analysis test was also completed, alongside the post hoc Tukey test (*p* < 0.05). The results of the changes in the expression of mRNA were presented as a fold change (FC) of the gene expression, compared to the control culture. In order to show which miRNAs were engaged with the expression regulation of selected mRNAs, the mirTAR database (http://mirtar.mbc.nctu.edu.tw/human/; accessed on 25 November 2020) was used. In the mRNA:miRNA interaction analysis, the miRTAR database, with the assumption of the following recommended parameters, Minimum Free Energy (MFE) ≤ −7 Kcal/mol, MFE ≤ −7 Kcal/mol, and alignment score ≥ 120, were used.

## 3. Results

### 3.1. The mRNA Microarray Profile of Genes Involved in EMT in the Tissue and Whole Blood Samples

Out of 22,273 mRNA, 226 mRNA were connected with the epithelial–mesenchymal transition. First, a single-factor ANOVA variance analysis was carried out with the utilization of the Benjamini–Hochberg correction, which indicated that, in samples obtained during hysterectomy, 60 mRNAs differentiated samples of endometrial cancer from the control (*p* < 0.05). Furthermore, to indicate the number of mRNAs differentiating the samples obtained from women with the given grade of histopathological advancement of endometrial cancer (G1–G3) from the control (C), the post hoc Tukey test was carried out. In the case of biopsies, the following numbers of differentiating mRNA were noted: G1 vs. C = 21, G2 vs. C = 19, and G3 vs. C = 10 (Table 3, *p* < 0.05).

In blood samples, 54 mRNAs were genes differentiating the samples obtained from patients with endometrial cancer from the control group (*p* < 0.05). The post hoc Tukey test allowed us to observe that G1 vs. C = 18, G2 vs. C = 14, and G3 vs. C = 9 (Table 4, *p* < 0.05).

**Figure 1:**
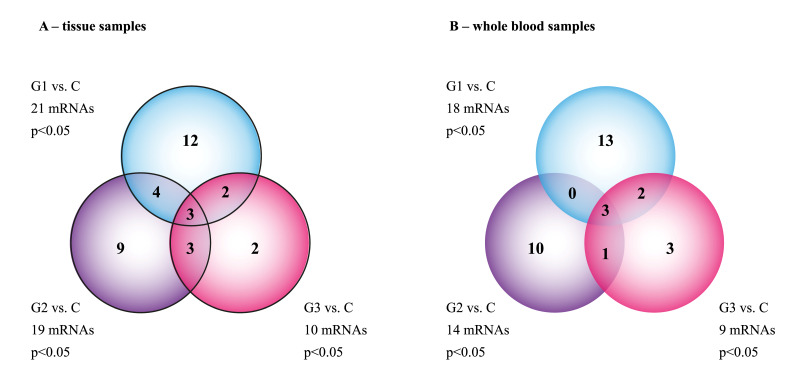
The Venn diagram of microarray results (*p* < 0.05). (**A**)—Tissue samples; (**B**)—whole blood samples.

In the next step of the analysis, a Venn diagram was constructed in order to identify genes common to all the transcriptome groups and characteristic only for a particular group (Figure 1).

The microarray analysis also showed that the common genes for the tissue and blood samples (−3.0 < FC > 3.0) were G1 vs. C: *TGFβ1* (Transforming growth factor isoform 1), *WNT5A* (vimentin 5A), *TGFβ2* (transforming growth factor isoform 2), and *NOTCH1*; G2 vs. C: *BCL2L*, *SOX9*, *BAMBI* (bone morphogenic protein and activin membrane-bound inhibitor), and *SMAD4*; G3 vs. C: *STAT1* and *TGFBβ1*. Additionally, mRNA *TGFβ1*, *NOTCH1*, and *BCL2L* are all common for all grades of endometrial cancer (Table 5; *p* < 0.05). Names of all transcripts of genes associated with the EMT process, differentiating the en-dometrial cancer tissue or blood samples G1-G3 in comparison to a control (*p* < 0.05) can be found in the Supplementary Materials accompanying this article.

### 3.2. Results of Real-Time Quantitative Polymerase Chain Reaction

**Figure 2:**
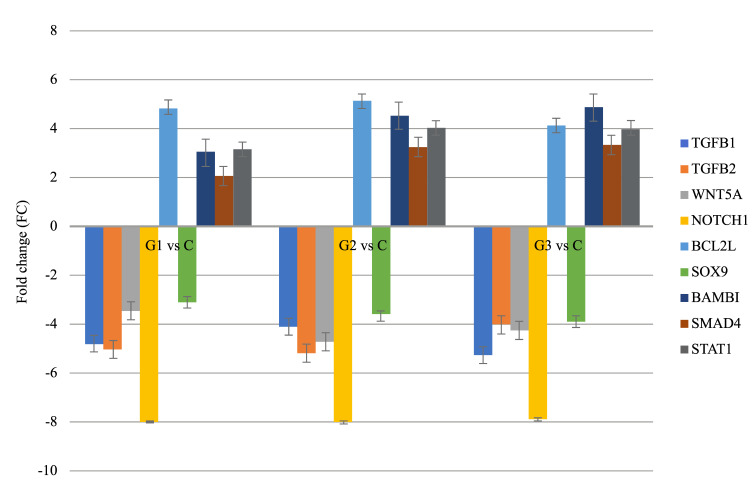
Expression pattern of genes related to EMT, which differentiates in patients with endometrial cancer (tissue, whole blood), compared to a control group.

In the next stage of molecular analysis, the real-time quantitative polymerase chain reaction (RTqPCR) was performed. The results obtained via RTqPCR showed the same direct course of changes in gene expression as the microarray analysis (Figure 2).

### 3.3. Results of miRNA Microarray

The following stage of molecular analysis was the assessment of the miRNA transcriptome in endometrial cancer samples and also in whole blood obtained from oncological patients, compared to a control. Out of 1105 human-specific miRNAs, 32 miRNAs differentiated the endometrial cancer tissue in a statistically significant way compared to the control, whereas, for blood samples, the number of miRNAs specifically differentiating the samples totaled 28 (−3.0 < FC > +3.0; *p* < 0.05). Afterward, similarly to the mRNA microarray analysis, the post hoc Tukey test was carried out, which, in the case of endometrial cancer tissue, indicated the following number of differentiating miRNAs at each given grade of histopathological differentiation from the control: G1 vs. C = 11, G2 vs. C = 10, and G3 vs. C = 4. For blood samples, it was noted that G1 vs. C = 15, G2 vs. C = 14, and G3 vs. C = 3. Analysis indicated that a change in the expression of miR-144, miR-106a, and miR-30d, which regulate the expression of genes connected with EMT, was statistically significant in the endometrial tissue and blood of patients. If the value of FC is higher than 1.0, it means that, compared to the control culture, there is an overexpression of genes, whereas, if the FC value is lower than 1.0, compared to the control, then there is a downregulation of genes. (Table 6).

### 3.4. Predictive Analysis of Expression Regulation of Genes Connected with EMT Selected as Differentiating through the miR-144, miR-106a, and miR-30d Molecules

**Figure 3:**
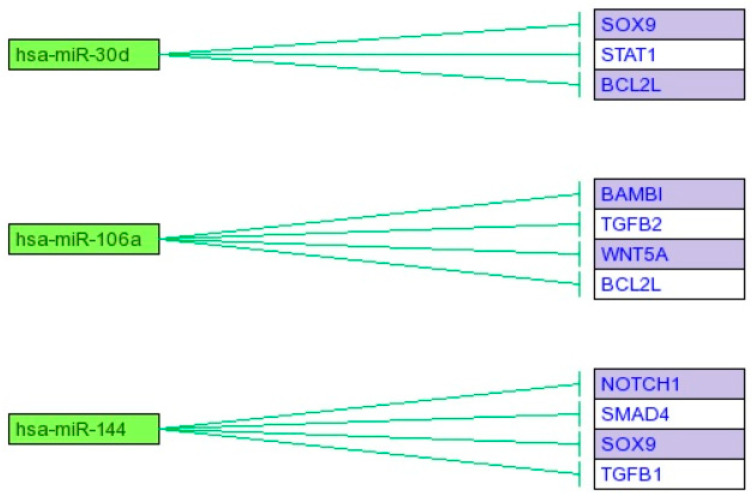
Predictive analysis of expression regulation of selected genes connected with EMT through miR-144, mir-106a, and miR-30d.

Next, using the mirTAR tool, the expression of which of the selected genes (Table 5) that could be potentially regulated by miR-144, miR-106a, and miR-30d (Figure 3) was determined.

### 3.5. Concentration of TGFβ1, NOTCH1, and BCL2L Determined by the Western Blot Technique

**Figure 4:**
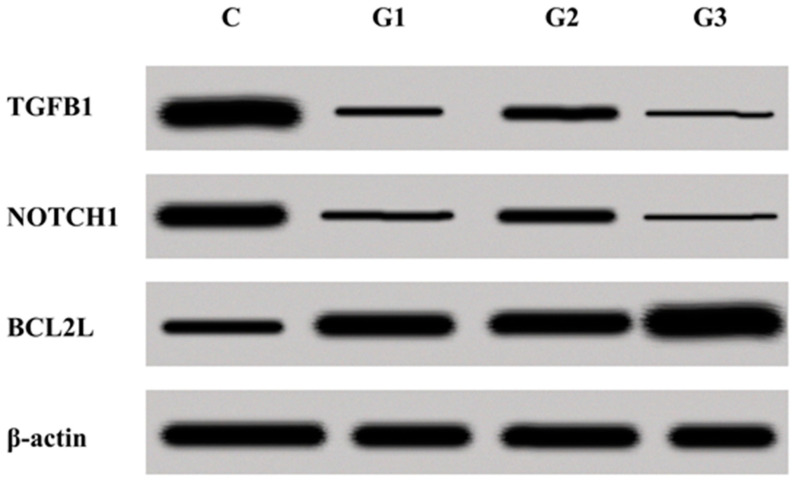
Expression of TGFβ1, NOTCH1, and BCL2L in endometrial cancer tissue (G1–G3) and a control obtained via the Western blot technique.

In the final stage, the expression changes of TGFβ1, NOTCH1, and BCL2L on the protein level were assessed within G1–G3 endometrial cancer specimens in comparison to the control (Figure 4).

## 4. Discussion

The aim of modern diagnostics is to select new molecular markers connected with the given disease as well as the processes that play a key role in the induction and development of that disease [25,26]. Such a methodology allows for changes on the molecular level to be noticed, which appear before phenotypic changes. Consequently, through early detection of abnormal changes, the chance for the implementation of effective therapy improves, which translates into the obtainment of disease remission as well as increases the five-year survival percentage in oncological illnesses [27]. However, attention has also recently been paid for modern diagnostics to not only be precise but also to not be invasive and to not cause discomfort to the patient [28,29].

We observed there were no references about the new trending molecular reclassification proposed by The Cancer Genome Atlas Research Network (TCGA). The new reclassification divides endometrial cancers into four distinct molecular subgroups, related to the prognosis, namely, ultramutates, hypermutates, low copy number, and high copy number. The ultramutated subgroup mostly consists of high-grade endometrioid endometrial carcinomas, presenting a very high mutational frequency, with mutations in the Polymerase-ε (POLE) exonuclease domain, and a good prognosis. The hypermutated subgroup includes endometrioid endometrial carcinomas, which have microsatellite instability and high mutational frequency of variable degrees, with an intermediate prognosis. The low copy number category contains low-grade endometrioid endometrial carcinomas, having a low mutational frequency with a good to intermediate prognosis. The high copy number subgroup mainly consists of serious endometrial carcinomas with a low mutational frequency, but a high rate of somatic copy number alterations, TP53 mutations, and a poor prognosis.

Post-surgical adjuvant treatment for women with aggressive cancers could be impacted by this reclassification of endometrial cancer [30].

Due to this, as part of this work, we analyzed changes in the expression profile of genes as well as miRNA molecules connected with EMT in the blood of women as well as in endometrial cancer G1–G3 samples alongside a control. Additionally, we attempted to determine if common mRNA and miRNA transcripts exist that differentiate test samples from the control in endometrial cancer tissue and whole blood.

As mentioned, phenotypic changes in immobile epithelial cells to that of mobile mesenchymal cells is a key process in metastasis of primary cancerous lesions.

EMT is a complex process, in which many different signaling pathways participate. The best-described signaling cascades connected with the discussed process include transforming growth factor beta (TGFβ) pathway, receptor tyrosine kinase (RTK)-dependent pathways, Nuclear Factor kappa-light-chain-enhancer of activated B cells (NF-κB) pathway, Wingless-related integration site (Wnt), Notch, and Hedgehog [31].

Based on genetic ontology, genes identified as differentiating in the microarray analysis belonged to the TGFβ pathway (TGFβ1, TGFβ2, SMAD4, BAMBI), Wnt (WNT5A, SMAD4), and Notch (NOTCH1) as well as apoptosis (BCL2L) [32].

TGF-β is the main inducer of the epithelial–mesenchymal transition, an inhibitor of proliferation in normal epithelial cells, halting them in the G1 phase of the cell cycle [33]. Currently, it is evident, however, that in neoplastically changed cells, TGFβ plays the role of a tumor progression promoter as well as the formation of distant metastatic foci [34].

Signalization dependent on TGF-β plays a double role in carcinogenesis and metastasis. During progression, the activity of TGFβ as an inhibitor of proliferation is halted, whereas the EMT process is promoted. This phenomenon has been named the TGFβ paradox. This cytokine is biologically active through Smad-dependent receptors or by activating pathways dependent on TGFβRI–III receptors [35,36].

Signal transduction dependent on TGFβ begins through its binding with three receptors: TGFβRI–III. The binding of TGFβ with TGFβRIIII allows for the recruitment and activation of TGFβRI, which leads to the induction of canonical signalization dependent on SMAD2/3. Next, heterocomplexes with Smad2/3 and SMAD4 are transported to the cell nucleus, where they play the role of transcriptional factors affecting the expression of the STAT family proteins [37].

Consequently, the signalization of TGFβ in the neoplastic process is a reduction in the expression of epithelial markers (E-cadherin and cytokeratin) with the simultaneous overexpression of mesenchymal markers (vimentin, N-cadherin, and fibronectin) [38]. It is also worthwhile to remember the positive interaction of the TGFβ pathway with the Notch, Wnt/β-catenin, nuclear factor (NF)-kB, and RTK pathways, having a synergistic effect on EMT [37,38].

Microarray analysis indicated that, compared to the control, in the study group there was a decrease in the expression of TGFβ1, SMAD4 with a simultaneous overexpression of mRNA BAMBI, which is described as a negative regulator of the TGFβ signaling pathway. Additionally, Perlino et al. [39] indicated there is a significant decrease in the expression of TGFβ1 in endometrial cancer samples, in comparison to the control. They used the Northern blot method for the detection of changes in the expression profile of mRNA TGFβ1 [39]. The results obtained by us are compatible with the observations of Soufla et al. [40]. These researchers bring attention to the fact that the decreased transcriptional activity of TGFβ1 can be a useful marker for the progression of neoplastic changes [40]. In our research, also, it can be determined that, together with an increase in the histopathological differentiation of endometrial cancer, the expression of TGFβ1 is increasingly lowered (G3 > G2 > G1 > C). In turn, Delforce et al. [41] noted a statistically significant increase in the expression of TGFβ1 in endometrial cancer samples, compared to the control culture [34]. This indicates the fact that biological effects on TGFβ signalization are conditioned by the biological context as well as the microenvironment on the tumor mass.

In reference to the expression of the second gene in the samples obtained from patients with endometrial cancer differentiating the culture from the control, that is, mRNA SMAD4, the observations of Sarah et al. are interesting. They assessed the expression of the discussed gene using the immunohistochemical (IHC) staining technique in gynecological tumors: ovarian cancer tumors; endometrial carcinoma; origin cervical, carcinoma, metastatic; and adenocarcinomas to ovary. They indicated that the silencing of SMAD4 expression is characteristic of the active process of metastasis of primary neoplastic lesions [42]. In addition, Giglio et al. noted a decrease in the expression of SMAD4 in endometrial cancer indicating that the protein coded by the SMAD4 gene plays a key, central role in the signalization of TGFβ [43].

The signalization of Wnt also plays a significant regulatory role in EMT through the canonical pathway (β-catenin) or via the non-canonical pathway. After receiving signals, Wnt, LPR5/6, and Fz form a complex, which influences the stabilization of β-catenin and its translocation to the cell nucleus, where it accumulates. The β-catenin forms a complex with a T-cell factor/lymphoid enhancer factor (TCF/LEF) factor, which initiates the transcription of Wnt target genes, including Snail1. During EMT, Smad2 and Smad4 influence the Wnt signal to eliminate the expression of E-cadherin in epithelial cells [44]. Based on the research conducted as part of this study, it was observed that there is a silencing in the expression of WNT5A, compared to the control, in endometrial cancer samples. Wasniewski et al. also noted the same expression pattern for the WNT5A mRNA and protein. Similar to the case of TGFβ, also in reference to WNT5A, it was indicated that the expression profile is specific to the tissue and can be dependent on the type of cancer [45]. Moreover, it should also be considered that the findings of Bauera et al. [46], who determined that, in the case of cervical cancer, the nature of WNT5a in the role of cancer development, whether by inhibition or promotion, depended on the WNT5A isoform, which has differing biological effects [47]. Additionally, it is highlighted that a decrease in the expression of WNT5A was positively correlated with the cell potential for metastasis and a worse prognosis [48].

Furthermore, it is suggested that the signalization of Wnt/β-catenin plays a significant role in the reprogramming of normal endometrial cells into tumor cells [49].

Another significant signaling pathway in the regulation of EMT is the Notch pathway, which is activated through the interaction between the Notch receptors and the ligands of neighboring cells. This pathway is responsible for keeping the balance between the proliferation of cells, differentiation, and apoptosis, in order to maintain the progenitor cell population. Notch signalization required coordination with other signals to promote EMT. TGF-β increases the activity of Notch through Smad3, by promoting the expression of Slug, which then halts E-cadherin. Alongside the epithelial–mesenchymal transition induced by Slug, there is the activation of β-catenin and anoikic resistance. It was indicated that between Wnt and Notch there are interactions to produce the neoplastic phenotype [50,51]. In our study, we determined that NOTCH1 differentiates the samples obtained from endometrial cancer patients compared to the control group, including a decrease in the expression of the discussed gene being noted, which codes the NOTCH1 protein. Like before, it also seemed that the expression of NOTCH1 was tissue-specific and depended on the type of cancer. In the case of tongue cancer, an increase in the expression of mRNA NOTCH1 was observed [51], whereas our analysis indicated the opposite result. Jonusiene et al., like us, determined that there is a significant decrease in the expression of mRNA NOTCH1 in endometrial cancer cell samples, in comparison to the control culture [52]. However, on the proteome level, these researchers, using the Western blot method, observed the lack of a significant difference in the concentration of the NOTCH1 protein in the samples obtained from endometrial cancer patients compared to the control [53]. This may suggest that the expression of NOTCH1 is variable depending upon the population and individual.

The conducted microarray analysis of the expression profile of genes connected with EMT indicated that mRNA BCL2L differentiates the endometrial cancer samples, independent of the histopathological grade of differentiation from the control. The protein coded by the BCL2L gene is a strong inhibitor of apoptosis and a protooncogene. It was observed that the overexpression in samples obtained from the study group confirmed its biological role [54,55]. It also confirmed our previous observations that the expression of BCL2L, to a small degree, is regulated by miRNA molecules, although the predictive analysis indicated something else. However, the fact should also be noted that between mRNA BCL2L and miR-30d or miR-106a there is complementarity; thus, the bond strength may be relatively low [56]. The expression profile of mRNA TGFβ1, NOTCH1, and BCL2L was the same on the protein level, which was indicated through the Western blot technique.

In the second part of the experiment, we attempted to determine whether the EMT process is regulated by miRNA as well as whether or not the selected miRNAs can affect gene expression, which differentiated the samples (blood and tissue) obtained from patients with endometrial cancer from the control group.

Microarray analysis indicated that miR-30d, miR-106a, and miR-144 have the strongest connection to EMT in endometrial cancer. Furthermore, it was observed that the expression of one mRNA can be regulated by more than one miRNA.

So far, miRNA molecules could be described, above all, as negative regulators of gene expression [57], whereas newer studies indicate that they can act as strengtheners of expression [58].

In the case of breast cancer, an increase in the expression of miR-20d was noted, which is described as an inhibitor of programmed cell death and a promoter of cancer cell proliferation. Furthermore, Han et al. indicated that miR-20d is a significant regulator of signaling pathways, in which the STAT family proteins participate [59]. In turn, Ye et al. [53] indicated that miR-30d acts as a proliferation suppressor in ovarian cancer through the blocking of EMT induced by TGFβ1 [60]. Our observations regarding the expression of miR-30d are compatible with the observations of Özcan [61], who indicated that miR-30d is a strong regulator of EMT during pancreatic cancer, through a decrease in the expression of mesenchymal markers [61]. Moreover, we observed an increase in the expression of miR-106a and miR-144 in the endometrial cancer samples. In the experiments of Tang et al. [62], they noted, similarly to us, an overexpression of miR-106a in endometrial cancer samples in comparison to the control, indicating that this miRNA molecule is engaged in the growth of the tumor, cell migration, and the formation of metastases [62]. The last of the selected miRNA is miR-144, which is engaged in the expression regulation of TGFβ1, NOTCH1, SMAD4, and SOX9, key in the EMT process [63].

This study also indicated the possibility of using fluid biopsy in the diagnosis of endometrial cancer. The potential aims include the evaluation of the mRNA expression profile, such as p53, the assessment of the DNA methylation pattern, EMT markers, apoptosis, and cell migration [64].

The strengths of this study include determination of the expression of genes connected to EMT on both the transcriptome and proteome levels and conduction of predictive analysis of the interaction of miRNA with mRNAs selected through the microarray experiment. Furthermore, according to our best knowledge, this is the first study in which it was attempted to correlate the results obtained from an endometrium obtained by hysterectomy with the results obtained, when the material used was whole blood.

Of course, the analysis conducted by us and the obtained results require confirmation. Justifiably, increasing the sample size is required, from each stage of histopathological advancement, ranging from G1–G3, as well as the control. Confirming the observed changes in the miRNA transcriptome through the microarray technique is also significant. Another factor limiting the results obtained by us was the assessment of the concentration profile of the assessed proteins, but from the endometrial tissue, not blood, based on the Western blot technique. Of course, the use of other modern molecular diagnostic methods, such as the next-generation sequencing technique, is advisable.

## 5. Conclusions

The completed molecular analysis indicated that mRNA *TGFβ1*, *TGFβ2*, *WNT5A*, *TGFB2*, *NOTCH1*, *BCL2L*, *SOX9*, *BAMBI*, *SMAD4*, and *STAT1* as well as miR-30d, miR-144, and miR-106a are connected with the epithelial–mesenchymal transition in endometrial cancer and can be considered as supplementary, diagnostic molecular markers.

Selected mRNA and miRNA transcripts seem to be promising targets of molecular endometrial cancer targeted therapies. It is also worth highlighting that, in this work, we presented these mRNA and miRNA, differentiating samples of endometrial cancer G1–G3 from the control common for biopsy and whole blood, which constitutes a premise for further utilization of the fluid biopsy technique in differentiating the advancement stage of endometrial cancer, already before surgery, which will lead to more effective treatment. It seems that these results also can be used in the prognosis of endometrial cancer.

## Figures and Tables

**Table 1:** Characteristics of patients included in a control and a study group.

	C (*n* = 30 Cases)	G1 (*n* = 15 Cases)	G2 (*n* = 8 Cases)	G3 (*n* = 7 Cases)
Age (years)	65.36 ± 10.29	67.22 ± 8.04	68.4 ± 10.09	64.88 ± 12.02
Height (m)	1.63 ± 0.14	1.59 ± 0.08	1.62 ± 0.05	1.59 ± 0.04
Weight (kg)	72.99± 13.95	74.41 kg ± 11.79	85.77 ± 21.99	85.22 ± 13.11
BMI	28.77 ± 7.14—overweight	29.01 ± 3.14—overweight	36.15 ± 10.44—1st degree of obesity	33.18 ± 5.44—1st degree of obesity

C—control; G—grading; BMI—body mass index; average ± standard deviation.

**Table 2:** The nucleotide sequence of primers used to amplify genes differentiating the studies samples from the control via the Reverse-Transcription Quantitative Polymerase Chain Reaction (RTqPCR) reaction.

mRNA	Primer Sequence (Forward, Reverse)
*TGFβ1*	Forward: 5′-TGAACCGGCCTTTCCTGCTTCTCATG-3′ Reverse: 5′-GCGGAAGTCAATGTACAGCTGCCGC-3′
*TGFβ2*	Forward: 5′-TACTACGCCAAGGAGGTTTACAAA-3′ Reverse: 5′-TTGTTCAGGCACTCTGGCTTT-3′
*WNT5A*	Forward 5′-CGTTAGCAGCATCAGTCCAC-3′ Reverse 5′-ACGGCATCTCTCTTTCACCA-3′
*NOTCH1*	Forward 5′-ACTGTGAGGACCTGGTGGAC-3′ Reverse: 5′TTGTAGGTGTTGGGGAGGTC-3′
*BCL2L*	Forward:5′-GTAGTTGGAGATGAGTTCGAGATTC-3′ Reverse:5′-TTCATCGAAAACCTAAATAAAACGT-3′
*SOX9*	Forward 5′-TTGAGCCTTAAAACGGTGCT-3′ Reverse: 5′CTGGTGTTCTGAGAGGCACA-3′
*BAMBI*	Forward 5′-GCTGGAACTTGGTGCAAAAT-3′ Reverse: 5′GCCATTTTTCTCGGAATCAA-3′
*SMAD4*	Forward 5′-TTGCTTCCACTTGAATGCTG-3′ Reverse: 5′-CTTCAAAGGGGACACCAAAA-3′
*STAT1*	Forward 5′-CCGTTTTCATGACCTCCTGT-3′ Reverse: 5′-TGAATATTCCCCGACTGAGC-3′
*ACTB*	Forward: 5′-TCACCCACACTGTGCCCATCTACGA-3′ Reverse: 5′-CAGCGGAACCGCTCATTGCCAATGG-3′

**Table 3:** The number of mRNAs that were differentially expressed in endometrial tissue samples.

Group	C	G1	G2	G3
C	60	21 ^A^	19 ^A^	10 ^A^
G1	22	60	17 ^B^	24 ^B^
G2	12	44	60	21 ^C^
G3	31	32	22	60

C, control; G, grade of endometrial cancer. ^A^ G1, G2, G3 vs. C at *p* < 0.05. ^B^ G2, G3 vs. G1 at *p* < 0.05. ^C^ G3 vs. G2 at *p* < 0.05.

**Table 4:** The numbers of mRNAs that were differentially expressed in the whole blood samples from patients with endometrial cancer compared to a control.

Group	C	G1	G2	G3
C	54	21 ^A^	18 ^A^	9 ^A^
G1	20	51	14 ^B^	23 ^B^
G2	11	39	51	20 ^C^
G3	28	34	19	49

C, control; G, grade of endometrial cancer. ^A^ G1, G2, G3 vs. C at *p* < 0.05. ^B^ G2, G3 vs. G1 at *p* < 0.05. ^C^ G3 vs. G2 at *p* < 0.05.

**Table 5:** Expression profile of mRNAs related to Epithelial–Mesenchymal Transition (EMT) differentiating endometrial cancer from the control (−3.0 < FC > 3.0; *p* < 0.05).

Groups Compared	ID	mRNA	FC	Expression
G1 vs. C	203085_s_at	*TGFβ1*	−4.89	Decreased
	220407_s_at	*TGFβ2*	−4.71	Decreased
	205990_s_at	*WNT5A*	−3.98	Decreased
	218902_at	*NOTCH1*	−7.77	Decreased
G2 vs. C	207005_s_at	*BCL2L*	+5.04	Increased
	202935_s_at	*SOX9*	−3.11	Decreased
	203304_at	*BAMBI*	+3.66	Increased
	202527_s_at	*SMAD4*	+3.01	Increased
G3 vs. C	AFFX-HUMISGF3A/M97935_5_at	*STAT1*	+4.01	Increased
	203085_s_at	*TGFβ1*	−5.07	Decreased

C, control; G, grade of endometrial cancer; FC, fold change.

**Table 6:** Expression profile of miR-144, miR-106a, and miR-30d in endometrial tissue and whole blood of patients with endometrial cancer, compared to a control (presented as fold change).

	Tissue			Whole Blood		
	miR-144	miR-106a	miR-30d	miR-144	miR-106a	miR-30d
G1 vs. C	+8.21	+11.44	−5.22	+8.44	+10.14	−5.14
G2 vs. C	+9.18	+9.14	−7.11	+8.36	+9.88	−7.44
G3 vs. C	+9.04	+8.11	−8.01	+10.02	+7.99	−9.44

## Data Availability

The data used to support the findings of this study are included in the article. The data will not be shared because of the third-party rights and commercial confidentiality.

## Supplementary Materials

The following are available online at https://www.mdpi.com/article/10.3390/jcm10071520/s1, Table S1: Table S1. Transcripts of genes associated with the EMT process, differentiating the en-dometrial cancer tissue samples G1-G3 in comparison to a control (p < 0.05). Table S2. Transcripts of genes associated with the EMT process, differentiating the whole blood samples from patients with endometrial cancer G1-G3 in comparison to a control (p < 0.05).
