# Supplementary material for: Molecular Landscape of the Epithelial–Mesenchymal Transition in Endometrioid Endometrial Cancer
Source: J Clin Med. 2021 Apr 6;10(7):1520. doi: 10.3390/jcm10071520 (PMC8038735; doi:10.3390/jcm10071520)
Supplement: Supplementary file 1 [file jcm-10-01520-s001.pdf]

Supplementary Material

Table S1. Transcripts of genes associated with the EMT process, differentiating the endometrial cancer tissue samples G1-G3 in comparison to a control (p<0.05)

Groups compared	ID	mRNA	FC	Expression
G1 vs C	220407_s_at	*TGFβ2*	-4.71	Decreased
	205990_s_at	*WNT5A*	-3.98	Decreased
	218902_at	*NOTCH1*	-7.77	Decreased
	221633_at	*NCAPH2*	-2.85	Decreased
	221636_s_at	*MARC2*	-2.99	Decreased
	221613_s_at	*ZFAND6*	+3.04	Increased
	221627_at	*TRIM10*	+2.74	Increased
	221640_s_at	*PIDD1*	+1.66	Increased
	206213_at	*WNT10B*	-2.01	Decreased
	206226_at	*HRG*	-2.01	Decreased
	206943_at	*TGFBR1*	-2.11	Decreased
	207035_at	*SLC30A3*	+1.85	Increased
G2 vs C	207005_s_at	*BCL2L*	+5.04	Increased
	202935_s_at	*SOX9*	-3.11	Decreased
	203304_at	*BAMBI*	+3.66	Increased
	202527_s_at	*SMAD4*	+3.01	Increased
	213532_at	*ADAM17*	+2.66	Increased
	213563_s_at	*TUBGCP2*	-4.58	Decreased
	213586_at	*CDKN2D*	-3.66	Decreased
	206067_s_at	*WT1*	+2.01	Increased
	207595_s_at	*BMP1*	+1.58	Increased
G3 vs C	AFFX-HUMISGF3A/M97935_5_at	*STAT1*	+4.01	Increased
	213917_at	*PAX8*	-2.05	Decreased
G1, G2 vs C	201983_s_at	*EGFR*	-1.85	Decreased
	201984_s_at	*EGFR*	-1.45	Decreased
	202019_s_at	*LANCL1*	+2.00	Increased
	202020_s_at	*LANCL1*	+2.03	Increased
G1, G3 vs C	203085_s_at	*TGFβ1*	-4.89	Decreased
	204731_at	*TGFBR3*	+2.44	Increased
G2, G3 vs C	202310_s_at	*COL1A1*	-2.11	Decreased
	202311_s_at	*COL1A1*	-1.98	Decreased
	202312_s_at	*COL1A1*	-2.07	Decreased
G1,G2,G3 vs C	214630_at	*CYP11B2*	+2.55	Increased
	205174_s_at	*QPCT*	-3.54	Decreased
	205199_at	*CA9*	-2.11	Decreased

C, control; G, grade of endometrial cancer; FC- fold change

Table S2. Transcripts of genes associated with the EMT process, differentiating the whole blood samples from patients with endometrial cancer G1-G3 in comparison to a control ($p<0.05$)

Groups compared	ID	mRNA	FC	Expression
G1 vs C	220407_s_at	*TGFβ2*	-4.71	Decreased
	205990_s_at	*WNT5A*	-3.98	Decreased
	218902_at	*NOTCH1*	-7.77	Decreased
	204147_s_at	TFDP1	+3.69	Increased
	204158_s_at	TCIRG1	-2.69	Decreased
	204159_at	CDKN2C	+4.01	Increased
	204194_at	BACH1	-2.09	Decreased
	207309_at	NOS1	-2.54	Decreased
	207310_s_at	NOS1	-2.77	Decreased
	207328_at	ALOX15	+1.98	Increased
	208211_s_at	ALK	-1.57	Decreased
	208212_s_at	ALK	-1.69	Decreased
	208218_s_at	ACVR1B	-2.01	Increased
G2 vs C	207005_s_at	*BCL2L*	+5.04	Increased
	202935_s_at	*SOX9*	-3.11	Decreased
	203304_at	*BAMBI*	+3.66	Increased
	202527_s_at	*SMAD4*	+3.01	Increased
	208226_x_at	ADAM22	+2.98	Increased
	208268_at	ADAM28	+.3.44	Increased
	215119_at	MYO16	+2.01	Increased
	215170_s_at	CEP152	-1.44	Decreased
	215209_at	SEC24D	-2.65	Decreased
	215512_at	MARCH6	+2.11	Increased
G3 vs C	AFFX-HUMISGF3A/M97935_5_at	*STAT1*	+4.01	Increased
	207012_at	MMP16	+2.98	Increased
	207013_s_at	MMP16	+3.04	Increased
G1, G3 vs C	203085_s_at	*TGFβ1*	-4.89	Decreased
	209591_s_at	BMP7	-2.74	Decreased
G2, G3 vs C	210118_s_at	IL1A	+.3.69	Increased
G1,G2,G3 vs C	203567_s_at	TRIM38	+2.74	Increased
	203568_s_at	TRIM38	+1.66	Increased
	210735_s_at	CA12	-1.44	Decreased

C, control; G, grade of endometrial cancer; FC- fold change
